# Co-operation of BRCA1 and POH1 relieves the barriers posed by 53BP1 and RAP80 to resection

**DOI:** 10.1093/nar/gkt802

**Published:** 2013-09-05

**Authors:** Andreas Kakarougkas, Amani Ismail, Yoko Katsuki, Raimundo Freire, Atsushi Shibata, Penny A. Jeggo

**Affiliations:** ^1^Genome Damage and Stability Centre, DNA double Strand Break Repair Laboratory, University of Sussex, Brighton BN1 9 RQ, UK and ^2^Unidad de Investigación, Hospital Universitario de Canarias, Instituto de Tecnologías Biomédicas, Ofra s/n, 38320 La Laguna, Tenerife, Spain

## Abstract

In G2 phase cells, DNA double-strand break repair switches from DNA non-homologous end-joining to homologous recombination. This switch demands the promotion of resection. We examine the changes in 53BP1 and RAP80 ionizing radiation induced foci (IRIF) in G2 phase, as these are factors that restrict resection. We observed a 2-fold increase in the volume of 53BP1 foci by 8 h, which is not seen in G1 cells. Additionally, an IRIF core devoid of 53BP1 arises where RPA foci form, with BRCA1 IRIF forming between 53BP1 and replication protein A (RPA). Ubiquitin chains assessed using α-FK2 antibodies are similarly repositioned. Repositioning of all these components requires BRCA1’s BRCT but not the ring finger domain. 53BP1, RAP80 and ubiquitin chains are enlarged following POH1 depletion by small interfering RNA, but a devoid core does not form and RPA foci formation is impaired. Co-depletion of POH1 and RAP80, BRCC36 or ABRAXAS allows establishment of the 53BP1 and ubiquitin chain-devoid core. Thus, the barriers posed by 53BP1 and RAP80 are relieved by BRCA1 and POH1, respectively. Analysis of combined depletions shows that these represent distinct but interfacing barriers to promote loss of ubiquitin chains in the IRIF core, which is required for subsequent resection. We propose a model whereby BRCA1 impacts on 53BP1 to allow access of POH1 to RAP80. POH1-dependent removal of RAP80 within the IRIF core enables degradation of ubiquitin chains, which promotes loss of 53BP1. Thus, POH1 represents a novel component regulating the switch from non-homologous end-joining to homologous recombination.

## INTRODUCTION

DNA non-homologous end-joining (NHEJ) and homologous recombination (HR) represent the two major pathways for DNA double-strand break (DSB) repair. NHEJ occurs throughout the cell cycle; HR occurs only in S/G2 phase (1). HR also repairs one-ended DSBs at stalled/collapsed replication forks in S phase (2). Regulation between HR versus NHEJ is complex but critical for the maintenance of genomic stability after DSB generation. Although NHEJ must be avoided at one-ended DSBs, current evidence suggests that NHEJ repairs the majority of DSBs in G2 phase, but if NHEJ does not ensue, then resection occurs committing to repair by HR (3,4). Thus, depending on the situation, NHEJ is either avoided or there is a controlled switch from NHEJ to HR. Current evidence suggests that regulating resection, an early event in HR, represents a critical step determining the commitment to HR (4).

The DNA damage signalling response (DDR) to DSBs involves the orchestrated assembly of DDR proteins at the DSB site (5,6). The MRE11/RAD50/NBS1 (MRN) complex recruits Ataxia and telangiectasia mutated protein (ATM), which phosphorylates H2AX aiding recruitment of the mediator protein, MDC1, and tethering of MRN and ATM at the DSB. The ubiquitin ligases, RNF8 and RNF168, are then recruited (5). Subsequent generation or exposure of methylated histone residues aids the localization of 53BP1, another mediator protein (7,8). This assembly is visualized as ionizing radiation induced foci (IRIF) at DSBs. This occurs in all cycle phases whilst another branch of recruited proteins that include BRCA1, RAP80, ABRAXAS, and BRCC36, form either uniquely or more robustly in G2 phase (9). The recruitment of these latter proteins is dependent on RNF8-dependent ubiquitylation but independent of 53BP1. The 53BP1 has been described as a factor restricting resection and hence HR, and I-Sce1 reporter assays for HR have shown that small interfering RNA (siRNA) 53BP1 leads to enhanced HR (10). BRCA1, in contrast, supports HR with siRNA BRCA1 leading to a deficiency in HR (11). Strikingly, loss of 53BP1 relieves the requirement for BRCA1 for HR, suggesting that a major role of BRCA1 is to overcome a barrier to resection posed by 53BP1 (12–14). Supporting a model of this nature, a recent study proposed that BRCA1 promotes HR by excluding 53BP1 to the IRIF periphery, thereby overcoming 53BP1’s inhibitory barrier on HR (15).

A complex encompassing RAP80, BRCC36 and ABRAXAS represents another factor that inhibits resection and promotes NHEJ (16–18). Indeed, RAP80 siRNA leads to unbridled resection and enhanced HR. RAP80 has a tandem ubiquitin interaction motif allowing it to bind to Lys^63^-linked ubiquitin polymers (17,19). Thus, RAP80 binds to Lys^63-^linked ubiquitin residues, which arise at IRIF as a consequence of the ubiquitin ligases, RNF8 and RNF168. It has been proposed that RAP80 serves to inhibit resection by binding to ubiquitin chains (17,18). POH1, a deubiquitylating enzyme (DUB) and component of the proteasome, has recently been shown to regulate 53BP1 via an ability to counteract RNF8/RNF168 dependent ubiquitin activity (20).Consequently, 53BP1 foci are enlarged in cells depleted for POH1. Additionally and distinctly, POH1 has a role in HR promoting the loading of RAD51.

Here, we examine changes in IRIF that arise in G2 cells during the switch from NHEJ to HR. Using Z-stacked immunofluorescence imaging and 3D reconstruction, we show that 53BP1 and ubiquitin chains but not γH2AX undergo a G2 phase-specific enlargement via a process requiring BRCA1’s BRCT domain. Additionally, BRCA1 promotes the formation of a core devoid of 53BP1 and ubiquitin chains in G2 phase in which RPA foci arise. As reported previously, 53BP1 foci are enlarged in G1 and G2 phase following POH1 depletion, but we show here that POH1 has a distinct role in generating a core devoid of 53BP1 and ubiquitin chains in G2 cells, thereby promoting RPA foci formation (20).The requirement for POH1 for generation of a devoid core can be relieved by depletion of any member of the RAP80/BRCC36/ABRAXAS complex. Thus, POH1 relieves a barrier to resection posed by RAP80. We show that RAP80 and 53BP1 represent distinct but interfacing barriers to resection. We propose a model whereby BRCA1 impacts on 53BP1 to allow access of POH1 to the ubiquitin chains, which in turn facilitates the removal of 53BP1, relieving the barrier to resection.

## MATERIALS AND METHODS

### Cell culture and irradiation

A549 and mouse embryonic fibroblast (MEF) cells were cultured in Dulbecco’s modified Eagle’s medium with 10% Foetal Calf Serum (FCS), l-glutamine, penicillin and streptomycin at 37°C in a humidified 95% air and 5% CO2 atmosphere. Cells were irradiated by exposure to a ^137^Cs source. For G2 experiments, aphidicolin (3 μg/ml) was added to cells before IR to prevent S-phase cells from progressing into G2. Unless otherwise stated, all results represent the mean and SD of three experiments.

### siRNA knockdown conditions

siRNA-mediated knockdown was achieved using HiPerFect Transfection Reagent (Qiagen, Hilden, Germany) following the manufacturer’s instructions. siRNA duplexes were transfected into 2 × 10^5^ of logarithmically growing cells per condition. Cells were then grown for 72 h before IR. BRCA1 (5′-GGAACCUGUCUCCACAAAG-3′) POH1 (5′- AGAGUUGGAUGGAAGGUUU-3′) and Abraxas (5′-CATCGACTGGAACATTCCTTATATA-3′) siRNA oligonucleotides were StealthTM RNAi oligos from Invitrogen. BRCA1, Artemis, BRCA2, BRCC36, RAP80, POH1, KAP-1 and CtiP siRNA oligonucleotides were obtained from the Dharmacon SMARTpool. The full list of target sequences from the Dharmcacon ON-TARGETplus SMARTpool is provided in Supplementary Figure S6B.

### Immunofluorescence

Cells plated on glass slides were fixed for 10 min with fixative [3% (w/v) paraformaldehyde (PFA), 2% (w/v) sucrose, 1× PBS] and permeabilized for 1 min with 0.2% Triton X-100 in PBS. When staining for RPA and RAD51 foci, pre extraction was performed by treatment with 0.2% Triton X-100 in PBS for 0.5–1 min before PFA fixation. Cells were rinsed with PBS and incubated with primary antibody diluted in PBS + 2% (w/v) BSA for 1 h at room temperature (RT). Cells were washed three times, incubated with secondary antibody [diluted in PBS+2% (w/v) BSA] for 30 min at RT in the dark, incubated with 4′,6-diamidino-2-phenylindole (DAPI) for 10 min and washed three times with PBS. In cases where quadruple staining was used (Figure 2), the cells were initially incubated with three compatible antibodies, followed by staining with their corresponding secondary antibodies. The cells were then sequentially stained with a fourth antibody and subsequently stained with a compatible secondary antibody. In all cases, slides were mounted using Vectashield and visualized/analysed using a Nikon-e400 microscope and imaged using an Applied Precision® Delta Vision® RT Olympus IX70 deconvolution microscope and softWoRx® Suite software. In each sample, a minimum of 30 cells was scored blindly, and error bars represent the SD between three experiments. Co-localization analysis (S2B) was undertaken using softWoRx® Suite software. The Pearson Coefficient of Correlation indicates how closely two intensities co-localize on a pixel-by-pixel basis (full co-localization is 1.0).

### Z stack imaging and 3D modelling

Z-stack imaging was carried out using an Applied Precision® Delta Vision® RT Olympus IX70 deconvolution microscope and softWoRx® Suite software. Z-stacks were set at 2 µm, and individual nuclei was imaged using 100× magnification. Deconvolution was performed by the Huygens Professional image processing software package (PSF- Theoretical, Max iterations- 400, Quality change threshold- 0.01). The 3D model conversions of these images using the softWoRx® Suite are displayed (Figures 2 and 4) and were subsequently magnified and displayed either as solid or wireframe structures. Wireframe images allow visualization of the inner regions of IRIF. Line plots (Figures 2–5) were performed by the softWoRx® Suite software on a minimum of 10 cells from each of three independent experiments. Error bars represent the SD.

### Foci volume quantification

Z-stack images were acquired and deconvolved as described earlier in the text. IRIF volume quantification was subsequently undertaken using the ‘surface’ tool of the BitPlane Imaris image processing software. In each sample, a minimum of 10 cells were analysed from each of three independent experiments. Error bars represent the minimum and maximum valid values. Maximum valid values, determined as the highest datum still within the 1.5 interquartile range of the upper quartile, were used to eliminate abnormally large values arising from foci ‘clumping’ and resolution limitations. Statistical analysis was carried out using the Mann–Whitney Rank Sum test. Data were deemed to be significant when a *P* < 0.05 was obtained.

### Antibodies

The primary antibodies used were as follows: γH2AX (mouse) and 53BP1 (mouse) (Upstate Technology, Billerica, USA) at 1:800, 53BP1 (rabbit) (Bethyl, Cambridge, England) at 1:800, RPA (rat) (Calbiochem, Billerica, USA) at 1:800, RPA (mouse) (Lifespan Biosciences, Suffolk, UK) at 1:100, Phospho RPA32 (S4/S8) (mouse) (Bethyl, Cambridge, UK), RAD51 (rabbit) (Santa-Cruz Biotechnology, Santa Cruz, USA) at 1:200, p-histone H3 (p-H3) Ser10 (mouse) (Upstate Biotechnology, Buckingham, UK) at 1:500; FK2 (mouse) (Millipore, Billerica, USA) at 1:400, RAP80 (rabbit) (Epitomics, Burlingame, USA) at 1:400. The antibodies against the human proteins BRCA1, RAP80 and MDC1 were raised in rabbits injected with the following antigens: amino acids 1350–1650 for BRCA1, 1–400 for RAP80 and 500–800 for MDC1. In all cases, the corresponding cDNA fragment was cloned into the pET-28 expression vector (Novagen, Billerica, USA) to express his-tagged fusion proteins in *Escherichia coli*. Once expressed, the proteins were purified using Ni-NTA resin (Qiagen, Hilden, Germany) following manufacturer’s instructions and were inoculated in rabbits to raise antibodies using standard procedures (Harlow and Lane, 1988).

The following secondary antibodies were as follows: FITC (Sigma Aldrich, Poole, UK) at 1:200, CY3 (Sigma Aldrich, Poole, UK) at 1:200, Alexa 488, Alexa647 and Alexa 555 (Invitrogen, Grand Island, USA) all at 1:400.

The RNF168-GFP construct was generated by Dr G. Stewart, University of Birmingham.

## RESULTS

### BRCA1 functions downstream of CtIP-dependent initiation of resection

**Figure 1.**
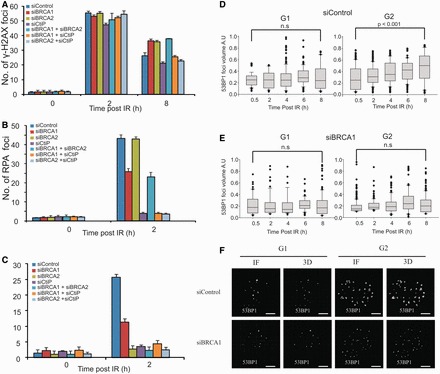
BRCA1 functions downstream of CtIP in G2 to promote resection and 53BP1 repositioning. (**A**) A549 cells were exposed to 3 Gy IR and γH2AX foci enumerated to 8 h post-IR. G2 cells were identified by CENPF staining (4). RPA (**B**) andRAD51 (**C**) foci in G2 phase A549 cells post 3 Gy IR following treatment with the indicated siRNAs. (**D** and **E**) Analysis of 53BP1 foci volume in A549 cells following 3 Gy IR in untreated cells (D) and following treatment with siRNA BRCA1 (E). (**F**) Representative images of nuclei immunostained for 53BP1 at 8 h after 3 Gy IR in G1 and G2 with or without siRNA BRCA1. Scale bars—5 μm. BRCA1 knockdown efficiency is shown in Supplementary Figure S1.

To monitor HR at two-ended DSBs and avoid the analysis of one-ended DSBs that can arise in S phase, we specifically examine irradiated G2 cells using the cell cycle marker, CENPF, and add aphidicolin to prevent S phase cells progressing into G2 during analysis. Extensive control experiments have shown that this does not impair DSB repair by either NHEJ or HR nor significantly increase the level of IRIF in undamaged cells (3,4). Two hours post 3 Gy IR, cells depleted for BRCA1 or BRCA2 show the same level of DSB repair, monitored by enumerating γH2AX foci, as control cells, consistent with previous findings that rapid DSB repair in G2 occurs by NHEJ (Figure 1A) (3,4). In contrast, a subtle but reproducible DSB repair defect is observed by 8 h, representing those DSBs repaired with slow kinetics by HR (Figure 1A). Longer times post-IR cannot be readily monitored by this procedure owing to mitotic progression. CtBP-interacting protein (CtIP) is required for initiating resection during HR (16). Previous studies have shown that siRNA CtIP prevents RPA, RAD51 and sister chromatid exchanges (SCE) foci formation but DSB repair occurs efficiently by NHEJ (4). Hence, it has been proposed that CtIP initiates resection, committing to repair by HR but if this initiation step does not occur, then repair can proceed by NHEJ. Consistent with this model, DSB repair after siRNA CtIP is dependent on XRCC4-like factor (XLF), an NHEJ factor (4). Consistent with the proposal that depletion of siRNA CtIP allows NHEJ to repair DSBs, we observed that siRNA CtIP rescues the DSB repair defect caused by siRNA BRCA2 in G2 cells (Figure 1A) (4).

In contrast to siRNA CtIP (where no DSB repair defect is observed in G2, siRNA BRCA1 does confer a G2 DSB repair defect identical to that shown by cells lacking essential HR components (RAD51 or BRCA2), suggesting that BRCA1 functions downstream of CtIP, likely at a stage post the commitment to HR (Figure 1A). To investigate the order of BRCA1 and CtIP function, we examined whether CtIP depletion could rescue the G2 repair defect caused by siRNA BRCA1. siRNA CtIP + BRCA1 relieved the defect observed following siRNA BRCA1 alone (Figure 1A). Thus, we propose that CtIP functions upstream of BRCA1 to initiate resection, committing to repair by HR. BRCA1, in contrast, functions to complete resection. As CtIP-dependent initiation of resection (the step committing to HR and precluding the usage of NHEJ) occurs in BRCA1-depleted cells, DSB repair cannot ensue by NHEJ. As neither HR nor NHEJ can proceed, a DSB repair defect is observed.

To substantiate this, we examined whether siRNA BRCA1 could rescue the repair defect in cells lacking BRCA2, a downstream HR factor required for RAD51 loading (21). We anticipated that if BRCA1 functions downstream of CtIP-dependent initiation of resection, siRNA BRCA1 would not rescue the BRCA2 repair defect, in contrast to the situation following siRNA CtIP. Indeed, although siRNA CtIP rescued the siRNA BRCA2 repair defect, joint siRNA BRCA1 + BRCA2 showed defective DSB repair (Figure 1A).

Extending the analysis of where BRCA1 functions, we examined RPA and RAD51 foci formation following siRNA CtIP. Consistent with other findings, siRNA BRCA1 causes a modest decrease in RPA and RAD51 foci numbers (Figure 1B and C) (22–24). Both endpoints are almost abolished following siRNA CtIP. As expected, siRNA BRCA2 does not impair resection (RPA foci numbers) but prevents RAD51 foci formation (Figure 1B and C) (4). Importantly, RPA and RAD51 foci numbers remained low following siRNA BRCA1 + CtIP or BRCA2 + CtIP. However, as shown in Figure 1A, DSB repair ensues following either siRNA BRCA1 + CtIP or BRCA2 + CtIP. Thus, we argue that CtIP initiates resection committing to repair by HR. In the absence of CtIP, DSB repair can ensue by NHEJ (4). As CtIP can rescue the DSB repair defect following siRNA of either BRCA1 or BRCA2 in the absence of detectable resection or RAD51 loading, we propose that both BRCA1 and BRCA2 function downstream of CtIP-dependent initiation of resection (Figure 1B and C).

Collectively, we interpret these findings as showing that CtIP’s upstream role in initiating resection commits to repair by HR. If prevented, DSB repair ensues by NHEJ (4). In contrast, siRNA BRCA1 confers a DSB repair defect with visible although diminished RPA foci. Thus, we propose that BRCA1 functions to complete resection downstream of CtIP’s initiation step. These findings are consistent with but extend previous findings that the resection observed in cells deficient for BRCA1 and 53BP1 is CtIP dependent (12).

### 53BP1 undergoes repositioning at IRIF during HR

**Figure 2.**
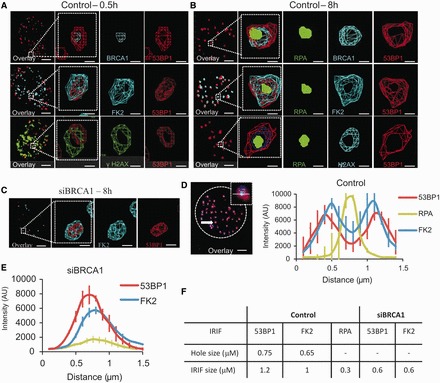
BRCA1 promotes 53BP1 repositioning in G2 creating a core devoid of 53BP1 and ubiquitin chains. (**A** and **B**) Analysis of G2 A549 cells at 0.5 (A) and 8 (B) h post 3 Gy IR. Following immunostaining with the indicated antibodies, 3D IRIF analysis was undertaken. The red and green signals represent 53BP1 and RPA, respectively; the blue signal is as indicated. Here, and in all cases where four antibodies were used, DAPI was omitted. This allowed us to use a blue secondary antibody (Alexa 350) for identifying G2 phase cells. A typical example would be as follows: Rabbit CENPF antibody coupled with a blue secondary antibody, rat RPA antibody coupled with a green secondary antibody, Rabbit BRCA1 antibody (these foci are also visible in the blue channel but do not interfere with pan-nuclear appearance of CENPF) coupled with a red secondary antibody, mouse 53BP1 coupled with a far red secondary antibody. ‘Wireframe’ images are displayed allowing 3D visualization. Foci volume enlarges from 0.5 to 8 h post-IR generating an expanded core lacking 53BP1. BRCA1 localizes internally to 53BP1 and RPA lies within the core**.** (**C**) Images following siRNA BRCA1 8 h post-IR. Scale bars—5 and 0.5 μm in magnified images. (**D** and **E**) Quantification of fluorescence intensity profiles along a line drawn through the centre of IRIF at 8 h post-irradiation. In control cells (D) by 8 h post-IR, 53BP1 and FK2 IRIF are distributed in a bipolar manner whilst in BRCA1 k.d cells, single peaks persist. A minimum of 10 cells were analysed from each of three independent experiments. Error bars represent SD. The 53BP1 and RPA foci intensity assessed in (E) represents an unbiased average of all IRIF after siRNA BRCA1 (i.e. those with or without detectable RPA foci). Analysis of IRIF with RPA foci is shown in Supplementary Figure S3. The 53BP1 repositioning was impaired to the same extent in IRIF with or without RPA foci after siRNA BRCA1. (**F**) Quantification of IRIF clearance and overall size in Control and BRCA1 siRNA-treated cells. IRIF clearance represents the distance between the peaks in IRIF that show a bipolar distribution, whereas the foci size was determined by measuring the distance between the outer edges of the peaks at 50% intensity. 53BP1 knockdown efficiency is shown in Supplementary Figure S1. The size of the RPA peak was not estimated after siRNA BRCA1 due to the lack of a defined peak.

BRCA1 has been proposed to overcome a barrier to HR created by 53BP1 in asynchronous cells (12,13). We tested whether BRCA1 has the same role at two-ended DSBs arising in G2 cells after IR exposure. We observed that in G2 cells, 53BP1 IRIF enlarge at later times (8 h) post-IR compared with IRIF in G1. We exploited Z-stacked immunofluorescence imaging and 3D reconstruction to quantify IRIF size and organization. In G1, no substantial change in IRIF volume post-IR using α-53BP1 was observed, whereas a doubling in 53BP1 foci volume was seen from 0.5 to 8 h in G2 (Figure 1D). Similar findings were recently reported, although the impact here is more marked most likely because we specifically examine two-ended DSBs in G2 (15). siRNA BRCA1 did not affect IRIF volume in G1 but precluded enlargement in G2, with IRIF volume resembling that observed in G1 (Figure 1E and F). Whereas 53BP1 localized as a tight sphere in G1 and at 30 min post-IR in G2, in striking contrast, it both vacated the central core and relocated to the periphery of enlarged IRIF by 8 h in G2 (Figure 2A and B). Strikingly, 53BP1 repositioning did not occur following siRNA BRCA1 (Figure 2C). Further, in control cells at 0.5 and 8 h post-IR, BRCA1 localized internally to 53BP1 (Figure 2A). RPA foci localized to the IRIF core that became devoid of 53BP1 with BRCA1 being located between 53BP1 and RPA (Figure 2B). RPA localization to the IRIF core is consistent with a report of a micro compartment of single-strand DNA-binding protein within the centre of micro-irradiated laser tracks (25).

### Ubiquitin chains but not γH2AX relocalize with 53BP1

53BP1 localizes to IRIF via RNF8-RNF168-dependent H2A ubiquitylation (26). We predicted that 53BP1 repositioning to IRIF periphery might necessitate enlargement of the region encompassing ubiquitin chains. Using 3D imaging with **α**-FK2 antibodies at 8 h post-IR in G2 cells, we observed that the IRIF core was depleted of the **α**-FK2 signal, which instead co-localized with 53BP1 (Figure 2A and B; Supplementary Figure S2B). Thus, FK2 co-localized with 53BP1 at 0.5 and 8 h post-IR in G2. In contrast, whereas γH2AX localizes externally to 53BP1 at 0.5 h post-IR, it does not relocalize to the same extent as 53BP1 and is positioned internally to 53BP1 by 8 h post-IR in G2 (Figure 2A and B; Supplementary Figure S2E). However, γH2AX does vacate the core where RPA foci form, although the devoid core is smaller than observed after 53BP1 (Figure 2B; for further details and discussion, see Supplementary Figure S2E and F).

To assess whether the inability of 53BP1 foci to enlarge in BRCA1 depleted cells might be a consequence of reduced resection (assessed as RPA foci numbers), we examined 53BP1 foci in cells depleted for the endonuclease Artemis, which is required for resection in G2 cells, as Artemis siRNA and BRCA1 siRNA result in a similar decrease in RPA foci numbers (3). Notably, we observed a similar 2-fold increase in 53BP1 foci volume in G2 cells to that seen in control cells, suggesting that BRCA1 directly promotes 53BP1 relocalization during HR rather than relocalization being an indirect consequence of impaired resection (Supplementary Figure S2C).

The aforementioned findings highlight two BRCA1-dependent changes in IRIF that can be visualized by 8 h post-IR compared with 0.5 h; first, the foci enlarge in volume, but additionally an enlarged core devoid of 53BP1 and ubiquitin chains arises. Quantification of the volume of IRIF at 8 h post-IR has been carefully monitored (Figure 1D and E). As an alternative way to quantitatively assess these changes and particularly to monitor the formation of a devoid core, we sought to monitor the distribution of DDR proteins at each IRIF along a linear axis (Figure 2D). This analysis shows that at 8 h post-IR in G2 cells, 53BP1 and ubiquitin chains are distributed in a bipolar manner displaying two peaks and a central trough. Significantly, RPA is distributed as a single peak locating to the 53BP1/ubiquitin chain trough. Moreover, a single peak distribution for both 53BP1 and ubiquitin chains is observed following depletion of BRCA1, and the intensity of the RPA signal is reduced (Figure 2E and Supplementary Figure S3). Further, the overall width of the outer territory occupied by 53BP1 or ubiquitin chains is ∼2-fold greater in control cells compared with that in cells treated with BRCA1 siRNA, consistent with the notion that BRCA1 regulates two processes, formation of a 53BP1-devoid core and enlargement of the 53BP1 foci (Figure 2F).

### BRCA1’s BRCT domain promotes 53BP1 repositioning and creation of a devoid core

**Figure 3.**
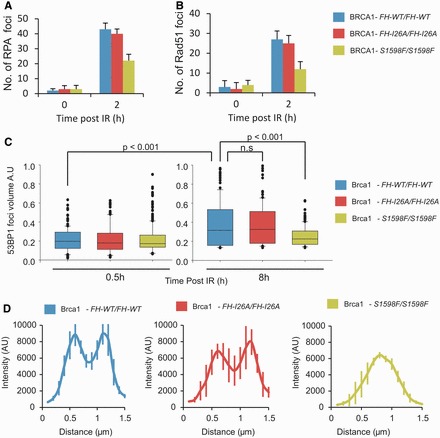
The BRCT but not the RING domain of BRCA1 is required for resection, RAD51 loading and 53BP1 repositioning during HR in G2. (**A**) *Brca1^FH-WT/FH-WT^*, *Brca1^FH-I26A/FH-I26A^* and *Brca1^S1598F/S1598F^* MEFs were irradiated with 3 Gy IR in the presence of aphidocolin. Cells were harvested 8 h post-IR and immunostained with DAPI, RPA and p-H3 antibodies to identify G2 cells. (**B**) As for (A), but RAD51 foci were enumerated 2 h post 3 Gy IR. (**C**) Analysis of 53BP1 foci volume in G2 phase *Brca1^FH-WT/FH-WT^*, *Brca1^FH-I26A^* and *Brca1^FH-S1598F^* MEFs 0.5 and 8 h following 3Gy IR. Analysis was carried out as in Figure 1C. (**D**) Quantification of the intensity of 53BP1 in G2 phase *Brca1^FH-WT/FH-WT^*, *Brca1^FH-I26A^* and *Brca1^FH-S1598F^* MEFs. Analysis was carried out as in Figure 2D. A minimum of 10 cells were analysed from three independent experiments. Error bars represent SD.

To examine the BRCA1 domains required for 53BP1 repositioning, we exploited MEFs homozygously expressing either *WT*, *Brca1*, *Brca1^FH-126A^*, which inactivates the E3 ligase activity, or *Brca1^S1598F^*, which disrupts the BRCT phospho-recognition domain (27). Analysis using these cells has shown that BRCA1’s BRCT domain is required for HR monitored by an I-Sce1 assay (27). Consistent with this, we observed that RPA and RAD51 focus formation is diminished in *Brca1^S1598F^* MEFs but occurs normally in *Brca1^FH^^-126A^* MEFs (Figure 3A and B). Furthermore, although foci expansion was less marked in MEFs compared with human cells, expansion was evident and occurs normally in MEFs expressing *WT* or *Brca1^FH126A^* but not in *Brca1^S1598F^* MEFs (Figure 3C). Assessment of the signal distribution as described earlier in the text revealed that disruption of BRCA1’s E3 ligase activity did not impact on the distribution of 53BP1 at the foci, whereas in contrast, a bimodal distribution failed to form when the BRCT domain is impaired (Figure 3D). The width of the IRIF size as assessed earlier in the text was also diminished in the BRCT mutant cells (Figure 3D). Thus, we conclude that BRCA1’s BRCT domain is required for 53BP1 repositioning and generation of a devoid core. These findings also support the role for BRCA1 in repositioning 53BP1 and generating a devoid core without relying on siRNA.

### POH1 is required for the generation of a devoid core of 53BP1 and ubiquitin chains via a process involving RAP80/BRCC36/ABRAXAS

**Figure 4.**
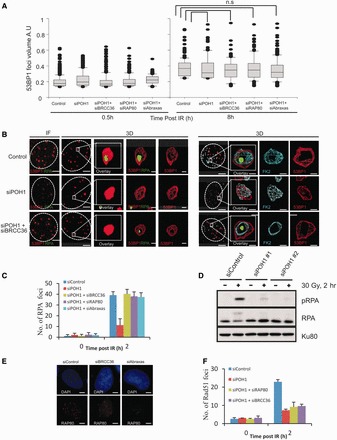
POH1 promotes resection in G2 by overcoming the inhibitory barriers of 53BP1 and RAP80. (**A**) 53BP1 foci volume was estimated in A549 cells treated with the indicated siRNA as in Figure 1C. (**B**) A549 cells were immunostained with the indicated antibodies 8 h post 3 Gy. The far left panels are a projection of the immunofluorescence (IF) Z-stacked images. The 3D model conversions of these images are shown in the subsequent panels. A devoid core of 53BP1 or FK2 does not form following siRNA POH1 but is visible following siRNA BRCC36 + POH1. A single oligonucleotide to POH1 was used, but similar results were obtained using a pool of siRNA POH1 oligonucleotides. RPA (**C**) and Rad51 (**F**) foci were enumerated in A549 cells treated with the indicated siRNA, and exposed to 3 Gy. (**D**) Resection was also assessed by monitoring phosphorylation of RPA on Ser 4 and 8 by western blotting. A549 cells were irradiated with 30 Gy following siRNA transfection, and whole-cell extracts were prepared at 2 h post-IR. (**E**) Depletion of BRCC36 or Abraxas results in loss of RAP80 demonstrating that they are required for RAP80 stability. Knockdown efficiency following siRNA POH1 is shown in Supplementary Figure S1. The 53BP1 foci size was assessed in all G2 cells following siRNA POH1, regardless of whether they have RPA foci or not. Scale bars—5 μm and 0.5 μm in magnified images.

**Figure 5.**
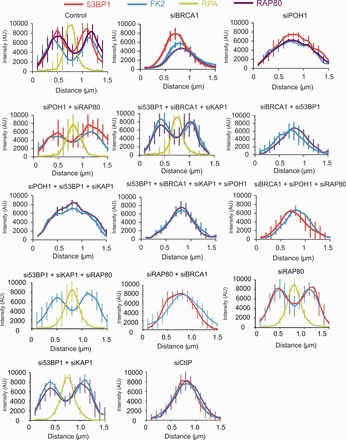
Analysis of IRIF clearance from resection sites in G2 phase following combined siRNA treatments. Clearance of 53BP1, RAP80 and Ubiquitin chains from the IRIF core is required for resection to proceed normally. Here, the distribution of these factors is demonstrated by quantification of the fluorescence intensity profiles along a line drawn through the centre of IRIF at 8 h post-irradiation (as in F). RPA foci intensity was not assessed in those situations where foci numbers were substantially (<50%) decreased due inaccuracy in assessing the average intensity of two distinct populations. The requirement of various factors for the clearance of 53BP1, RAP80 and Ubiquitin chains was analysed using combined siRNA-mediated knockdown. Knockdown efficiency following multiple siRNA transfection is shown in Supplementary Figure S1. A minimum of 10 cells were analysed from three independent experiments. Error bars represent SD. Quantification of IRIF clearance and overall size in cells treated with the various combinations of siRNA-mediated knockdown is shown in Supplementary Figure S6. The data for control cells and siRNA BRCA1 for 53BP1, ubiquitin chains and RPA foci is the same as that shown in Figure 2D and E. RAP80 analysis is additional.

BRCA1’s BRCT domain mediates interaction with CtIP, BACH1 and a complex encompassing RAP80-BRCC36-Abraxas (16). As CtIP initiates HR upstream of BRCA1, we considered it unlikely that CtIP is the factor mediating BRCA1 function in 53BP1 repositioning. Normal 53BP1 foci enlargement and repositioning was observed following siRNA RAP80, BRCC36 or BACH1 (Supplementary Figure S4A). As the process involves repositioning of ubiquitin chains, we reasoned that it might require a DUB, but surprisingly BRCC36 was not required (Supplementary Figure S4A). A recent study showed that the DUB, POH1, functions to restrict 53BP1 accumulation at IRIF by antagonizing RNF8/RNF168-mediated ubiquitination and promoting JMJD2A chromatin retention, which competes with 53BP1 for binding to dimethylated histone H4 residues (20). Hence, 53BP1 IRIF are enlarged at 1 h post 2 Gy IR following POH1 siRNA. We examined whether POH1 activity might have a function in regulating 53BP1 localization during the progression of HR in G2 cells. First, we assessed the size of 53BP1 foci following POH1 siRNA in G1 cells at times up to 8 h post-IR. The 53BP1 foci were enlarged at early times following POH1 siRNA, although by 8 h, they were of similar size to those in control cells (Supplementary Figure S5A). In G2 cells, 53BP1 foci were similar in size to those in control cells at 0.5 and 8 h post-IR (Figure 4A). These findings are consistent with the previous conclusion that POH1 activity antagonizes RNF8/RNF168-dependent ubiquitylation and restricts 53BP1 foci size (20). However, the enlargement of 53BP1 in G1 cells following POH1 siRNA makes it difficult to evaluate whether POH1 downregulation or restricted substrate access plays any role in the regulated enlargement of 53BP1 foci in G2 cells. Strikingly, however, although we observed 53BP1 foci expansion following POH1 siRNA, vacation of ubiquitin chains and 53BP1 from the core was greatly reduced, and RPA foci did not form in the majority of cells (Figure 4A and B). Consistent with this, RPA foci numbers were markedly reduced following siRNA POH1 (Figure 4C). Consolidating this, we observed that IR-induced RPA phosphorylation is POH1-dependent (Figure 4D). Quantification of the distribution of IRIF factors as described earlier in the text confirmed these findings (Figure 5). The distinction between siRNA BRCA1, which prevents expansion of 53BP1/ubiquitin chains and the formation of a devoid core versus siRNA POH1, which allows IRIF expansion but prevents devoid core formation, is further substantiated by this analysis. These findings strongly suggest that POH1 is required for the generation of a core in the IRIF devoid of 53BP1 and ubiquitin chains, which appears to be a prerequisite for a normal level of RPA foci formation.

Recent studies have proposed that RAP80 suppresses resection, as unbridled resection occurs following RAP80 siRNA (18). As depletion of RAP80, BRCC36 or ABRAXAS enhances resection, we examined the impact of combined POH1 and either siRNA RAP80, BRCC36 or ABRAXAS (17,18). In all cases, we observed normal 53BP1 enlargement but surprisingly a core devoid of ubiquitin chains and 53BP1 now formed (Figures 4A and B and 5). Moreover, RPA foci formed in the core with foci numbers returning to normal levels, although the foci were smaller than in control cells (Figure 4B and C). These findings were substantiated by the quantitative assessment of foci parameters as discussed earlier in the text (Figure 5 and Supplementary Figure S6). As RAP80 binds to ubiquitin chains, we considered it likely that the ability of siRNA BRCC36 or ABRAXAS to relieve the requirement for POH1 was caused by the ability of BRCC36 and ABRAXAS to interact with and stabilize RAP80 (18). Consistent with this possibility, we observed diminished expression of RAP80 following siRNA BRCC36 or siABRAXAS (Figure 4E).

siRNA POH1 also resulted in markedly reduced RAD51 foci numbers consistent with the dramatic reduction in RPA foci (Figure 4F). Unexpectedly, however, siRNA POH1 + BRCC36 did not restore RAD51 foci formation despite promoting recovery of RPA foci (Figure 4F). Thus, we conclude that POH1 has an additional function in RAD51 loading, which is likely distinct to its role in creating a 53BP1-devoid core to IRIF. This finding is consistent with a role for POH1 in RAD51 loading as reported previously (20).

### POH1 removes ubiquitin chains to promote loss of RAP80 and 53BP1 from the core

Our quantitative analysis of protein/factor distribution at the IRIF provided a means to assess changes to IRIF arising following a range of combined knockdown conditions. The ability of RAP80 to relieve the requirement for POH1 to create a vacant core suggests that RAP80 might be an additional factor that needs to be redistributed at the IRIF to promote resection. We, therefore, additionally examined the distribution of RAP80 at IRIF (Supplementary Figure S5B). Like 53BP1, in control cells, RAP80 vacates the core and is repositioned to the periphery of enlarged IRIF (Supplementary Figure S5B and Figure 5). This redistribution also requires BRCA1. To gain insight into the order of events, we carried out combined siRNA-mediated knockdown.

In previous work, we have shown that 53BP1 has a role in heterochromatin relaxation by serving to tether ATM at DSBs (28). This function is also required in G2 phase cells and confers a role for 53BP1 in promoting HR at HC-DSBs (manuscript submitted). To relieve this role of 53BP1 and focus on its inhibitory role in resection, we carry out siRNA KAP-1, which serves to relax the HC and overcomes the need for 53BP1 and ATM in DSB repair. siRNA KAP-1 does not impact on the rate of DSB repair in G2 phase cells or the IRIF enlargement and core generation (data not shown). Consistent with previous findings that loss of 53BP1 relieves the requirement for BRCA1 for HR, we observed that combined siRNA KAP-1, 53BP1 and BRCA1 restored the bipolar distribution of RAP80 and ubiquitin chains and allowed RPA foci to form in the core (although less efficiently than in control cells). The failure of these changes at IRIF to arise following siRNA 53BP1 + BRCA1 without siRNA KAP1 is consistent with our finding that HR does not proceed under these conditions (manuscript submitted). The fact that RAP80 redistribution is also regained raises the possibility that 53BP1 prevents access of POH1 to ubiquitin chains and/or RAP80 and that BRCA1 overcomes this block. Further, this analysis also revealed that although the distribution of 53BP1 and RAP80 was enlarged following siRNA POH1, neither 53BP1 nor RAP80 vacated the core region. Thus, both POH1 and BRCA1 are required to generate a core devoid of 53BP1, RAP80 and ubiquitin chains.

We next examined a range of additional situations; first, we co-depleted BRCA1, RAP80 and POH1 as well as BRCA1 + RAP80 but not POH1 (Figure 5 and Supplementary Figure S6). In both cases, 53BP1, as expected, remains within the IRIF core; additionally ubiquitin chains remain in the core. Thus, loss of RAP80 alone is insufficient to promote loss of the FK2 chains and BRCA1 function (to remove 53BP1) is additionally required. Next, we depleted 53BP1 (and KAP-1) and POH1. In this situation, we anticipated that RAP80 remains within the IRIF core and might prevent the loss of ubiquitin chains; indeed, ubiquitin chains retained a mono-polar distribution. Further consolidating this notion, combined depletion of 53BP1, BRCA1, KAP-1 and POH1 caused a monopolar distribution of ubiquitin chains. Interestingly, however, siRNA BRCA1 impacted on the width of the IRIF and reduced the enlargement otherwise observed following siRNA POH1 (see ‘Discussion’ section). Finally, we also examined combined 53BP1 (and KAP-1) and RAP80 depletion. In this case, both barriers limiting resection are removed, and we anticipated that normal foci enlargement and clearance would occur. As expected, the redistribution of ubiquitin chains occurred and RPA foci formed in a devoid core (Figure 5). As additional controls, we show that depletion of RAP80 alone or 53BP1 combined with KAP1 does not affect the redistribution of 53BP1 or ubiquitin chains. Further, the repositioning of IRIF components is not observed following siRNA CtIP, consistent with the notion that under this condition DSB repair occurs by NHEJ since resection is not initiated. In the situations shown in Figure 5, depletion of BRCA1 or POH1 resulted in a modest or very marked reduction in RPA foci formation, respectively, except when BRCA1 was co-depleted with 53BP1 or POH1 co-depleted with RAP80. In all situations where 53BP1 foci and ubiquitin chains are not repositioned, RPA foci formation is impaired (data not shown).

In conclusion, the creation of an IRIF devoid core and RPA foci formation requires both a BRCA1-dependent impact on 53BP1 plus a POH1-dependent impact upon RAP80. Failure to clear either 53BP1 or RAP80 prevents the clearance of ubiquitin chains and the ability to form RPA foci normally.

## DISCUSSION

Our findings reveal two distinct changes that take place at IRIF in G2 phase during the progression of HR; the enlargement of IRIF containing 53BP1, RAP80 and ubiquitin chains and the formation of a core devoid of these components. At early times in G2 phase, IRIF form in a similar manner to those in G1 phase, with 53BP1 appearing as tight foci overlapping with γH2AX. Subsequently and specifically in G2, BRCA1 promotes relocalization of all three components, 53BP1, RAP80 and ubiquitin chains to the periphery of enlarged IRIF. γH2AX is not, however, repositioned, although a core of reduced γH2AX intensity does form, although less marked than that observed for 53BP1. In parallel, RPA foci form in the devoid core. The IRIF volume enlargement represents a doubling in size. Although this could represent movement along the DNA molecule, the volume increase occurs in 3D (i.e. spherically) rather than longitudinally raising the possibility that it represents 53BP1 repositioning to encompass the undamaged sister homologue (see Supplementary Figure S7 for details of a model). This analysis of enlargement of 53BP1 foci is consistent with but extends a previous study showing that BRCA1 repositions 53BP1 to the foci periphery in S/G2 phase (15).

To facilitate analysis of these changes, we quantified the protein distribution at IRIF using 3D reconstruction and imaging analysis. As one approach, we monitored foci volume, which revealed the 2-fold increase described earlier in the text. Additionally, we monitored the distribution of DNA damage response proteins along an axis traversing the IRIF. Although there are limitations to this latter analysis, the quantification is fully consistent with our visual observations and the quantification of IRIF volume. Although this approach revealed the presence of a core with reduced occupancy of 53BP1 or ubiquitin modifications, the magnitude of reduction is potentially underrepresented. Indeed, the presence of a devoid core is more evident visually. Nonetheless, the marked distinction using the quantification approach allows an examination of factors regulating the process.

A previous study has reported that siRNA POH1 results in enlarged 53BP1 foci at 2 h post-IR in G1 phase cells and proposed that POH1 has a role in antagonizing RNF8-RNF168-mediated ubiquitylation. Our findings are consistent with this. Although the IRIF enlargement specific to G2 phase cells at 8 h post-IR occurred normally in POH1-depleted cells, it is unclear whether specific downregulation of POH1 activity or access underlies the regulated, G2-specific IRIF enlargement or whether it is an indirect consequence of POH1 siRNA as observed in G1 phase cells. Here, we focus on a distinct and novel role for POH1 in generating a devoid core region in IRIF. Our findings reveal the following features: (i) BRCA1 has a previously undescribed function in promoting clearance of RAP80 and ubiquitin chains during HR. This role, however, involves a previously described impact on 53BP1 (12–14). Indeed, BRCA1 is dispensable for clearance of RAP80 and ubiquitin chains in the IRIF core if 53BP1 is additionally depleted. (ii) POH1 promotes RAP80 clearance from the IRIF core but is not essential for the clearance of the ubiquitin chains, as a bipolar distribution of ubiquitin chains is regained following combined depletion of POH1 and RAP80. RAP80 binds to K63 linked ubiquitin chains via its tandem ubiquitin binding motif (17,18). It has been proposed that RAP80 protects the ubiquitin chains and that its loss leads to unbridled resection (17,18). Thus, we propose that POH1 facilitates the removal of RAP80, enabling degradation of the ubiquitin chains possibly via other DUBs. However, we cannot eliminate the possibility that POH1’s DUB activity degrades the ubiquitin chains, which promote RAP80 loss and that in the absence of RAP80, they become accessible to other DUBs that are not normally exploited in its controlled regulation.

53BP1 is known to be tethered at DSBs via its binding to H4K20Me2 groups (29). Significantly, the access of these residues appears to be regulated by RNF8/RNF168 dependent ubiquitylation of histone H2A and potentially by competition with binding of JMJD2 (7,8). A current model is that JMJD2 competes with 53BP1 for H4K20me2 binding and is prevented by ubiquitin chains. Thus, the loss of ubiquitin chains is likely a prerequisite for 53BP1 clearance; indeed, POH1 has been reported to regulate 53BP1 binding via its regulation of ubiquitin chains (20). However, it has also been proposed that valosin containing particle (VCP) regulates the unmasking of 53BP1 chromatin binding sites (7). Our finding here suggests that POH1 regulates 53BP1 binding and repositioning during HR via a process involving the removal of RAP80. Indeed, our findings show an unbroken relationship between the loss of ubiquitin chains and resection in the IRIF core; that is, the ubiquitin chains must be lost to allow normal resection. This process necessitates loss of 53BP1 and RAP80 from the core consistent with their previously described functions in restricting resection.

It is perhaps, noteworthy, that although POH1 depletion allows the IRIF to expand to a similar extent to that observed in control cells at 8 h in G2 cells, this impact is reduced when combined with BRCA1 siRNA. This effect cannot be attributed entirely to BRCA1’s impact on 53BP1, as it is also observed following co-depletion of BRCA1 and 53BP1. BRCA1 clearly has a role in promoting IRIF enlargement in G2 phase cells, which is likely distinct to its role in generating a devoid core. Our findings do not address whether downregulation of POH1 contributes to this process, and we surmise that the IRIF enlargement observed at 8 h in G2 cells following POH1 maybe a consequence of its previously described role in counterbalancing RNF8/RNF168-dependent ubiquitylation. It is possible, however, that BRCA1 specifically facilitates new RNF8/RNF168 ubiquitylation events during this G2-specific event.

Interestingly, our findings here reveal that 53BP1 enlarges in G2 phase without the concomitant enlargement of γH2AX. Previous studies have suggested that RNF168 can, via its RING domain and several ubiquitin-binding domains, amplify ubiquitin conjugates generated by its own activity and hence extend the region of histone ubiquitylation (30–33). This process appears to be restricted by several DUBs as well as by the ubiquitin ligases, TRIP12 and UBR5 (33). As a step towards revealing how 53BP1 might relocalize without γH2AX, we examined whether RNF168 also becomes relocalized. As the available RNF168 antibodies were not efficient for immunofluorescence, we expressed GFP-tagged RNF168. Significantly, in cells where 53BP1 foci were of similar size to those in control cells, we observed RNF168 relocalization to the foci periphery in a similar manner to 53BP1 (Supplementary Figure S4C). This suggests that RNF168’s ability to extend the region of ubiquitylation may underlie 53BP1 expansion. However, further studies are required to understand the detailed regulation of this step.

**Figure 6.**
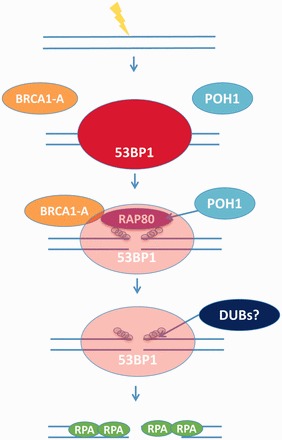
Model showing the removal of the inhibitory barriers posed to resection by 53BP1, RAP80 and Ubiquitin chains. Following the induction of DSBs, several factors including 53BP1, BRCA1 and RAP80 rapidly accumulate and form IRIF. The recruitment of 53BP1 and BRCA1 is dependent on the formation of ubiquitin chains at DSBs, which also form IRIF and can be visualized by antibodies that detect conjugated ubiquitin (a-FK2). Three complexes (BRCA1A-C) involving BRCA1 have been described (16). BRCA1-A comprises ABRAXAS, RAP80, BRCC36, BRCC45 and MERIT40. The presence of 53BP1, RAP80 and ubiquitin chains promote repair via NHEJ, as they are inhibitory to HR by blocking the process of resection. In G2 phase, the inhibitory barrier posed by these factors needs to be overcome for resection to proceed. Initially, BRCA1, via a process that requires its BRCT domain, ‘primes’ 53BP1 to allow POH1 access to RAP80. In the absence of BRCA1, both 53BP1 and RAP80 remain in the IRIF core. POH1 promotes the clearance of RAP80, which in turn allows removal of the ubiquitin chains from the IRIF core possibly via a DUB activity. This, in turn, promotes the full clearance of 53BP1 from the IRIF core. Thus, BRCA1 alone is not sufficient to promote clearance of 53BP1 from the core; POH1 is additionally required. Likewise, BRCA1 and POH1 are required to promote the clearance of RAP80 from the core. Once all these factors have been cleared from the IRIF core, nucleases can promote 5′-3′ resection, thus allowing repair by HR to proceed. We have proposed a linear feedback model to accommodate the finding that depletion of BRCA1 or POH1 can block clearance of both RAP80 and 53BP1, demonstrating that they are inter-dependent barriers. In the model shown, we have only depicted the removal of proteins/modifications from the IRIF core and have not included the repositioning of the proteins to the periphery of the foci.

Our findings demonstrate that 53BP1 and RAP80 represent two barriers to resection and that BRCA1 and POH1, respectively, function in an interfacing manner to overcome these barriers. Our data show that these are not simply alternative barriers, as one barrier is not relieved without the other and vice versa. We propose the following linear model for clearance of RAP80 and 53BP1 from the IRIF core (Figure 6). The process is initiated by a BRCA1-dependent ‘priming’ of 53BP1. As this requires BRCA1’s BRCT domain, we suggest that it could involve BRCA1’s modulation of 53BP1 position via binding to phosphorylated 53BP1. This process removes an inhibitory impact of 53BP1 on POH1, allowing it to access RAP80 and/or ubiquitin chains. POH1, as a component of the proteasome, may directly degrade RAP80, which allows access of ubiquitin chains to another DUB, or POH1 could remove the ubiquitin chains, which triggers loss of RAP80. As discussed earlier in the text, we favour the first possibility. Loss of the ubiquitin chains promotes binding of JMJD2, which promotes loss of 53BP1 by competitive binding to H4K20Me3 residues. Thus, BRCA1 initiates a cascade of events, which together with POH1 allow the formation of a core region in IRIF devoid of RAP80 and 53BP1, where RPA foci form. In summary, an emerging model for DSB repair in G2 phase cells is that NHEJ makes an initial attempt to repair DSBs, but resection and hence HR occurs when repair by NHEJ does not progress. The regulation of resection is a complex process that is critical to determine the choice between HR and NHEJ. At least two complexes, RAP80 and 53BP, function to prevent immediate DSB resection and allow NHEJ to attempt repair. We show here that resection requires the generation of a core region in IRIF devoid of 53BP1, RAP80 and ubiquitin chains and that RPA foci form in this devoid core. Both BRCA1 and POH1 are required for this process. We demonstrate a direct role for POH1 in clearing RAP80 in the IRIF core and an indirect role for BRCA1, which necessitates its impact on 53BP1.

## SUPPLEMENTARY DATA

Supplementary Data are available at NAR Online.

## FUNDING

The PAJ laboratory is supported by an MRC programme grant, the Association for International Cancer Research, the Wellcome Research Trust and the EMF Biological Research Trust. Spanish Ministry of Science and Innovation [SAF2010-22357 to R.F.]; and CONSOLIDER-Ingenio 2010 [CDS2007-0015 to R.F.]. Funding for open access charge: University of Sussex.

*Conflict of interest statement*. None declared.

## Supplementary Material

Supplementary Data
