# Supplementary material for: Co-operation of BRCA1 and POH1 relieves the barriers posed by 53BP1 and RAP80 to resection
Source: Nucleic Acids Res. 2013 Sep 5;41(22):10298–311. doi: 10.1093/nar/gkt802 (PMC3905848; doi:10.1093/nar/gkt802)
Supplement: Supplementary Data [file supp_41_22_10298__index.html]

Co-operation of BRCA1 and POH1 relieves the barriers posed by 53BP1 and RAP80 to resection — Co-operation of BRCA1 and POH1 relieves the barriers posed by 53BP1 and RAP80 to resection — Supplementary Data 

# Co-operation of BRCA1 and POH1 relieves the barriers posed by 53BP1 and RAP80 to resection

## Supplementary Data

files

**Files in this Data Supplement:**

- Supplementary Data - pdf file
- Supplementary Data - docx file
