# Supplementary material for: Co-operation of BRCA1 and POH1 relieves the barriers posed by 53BP1 and RAP80 to resection
Source: Nucleic Acids Res. 2013 Sep 5;41(22):10298–311. doi: 10.1093/nar/gkt802 (PMC3905848; doi:10.1093/nar/gkt802)
Supplement: Supplementary Data [file supp_gkt802_nar-00526-d-2013-File009.pdf]

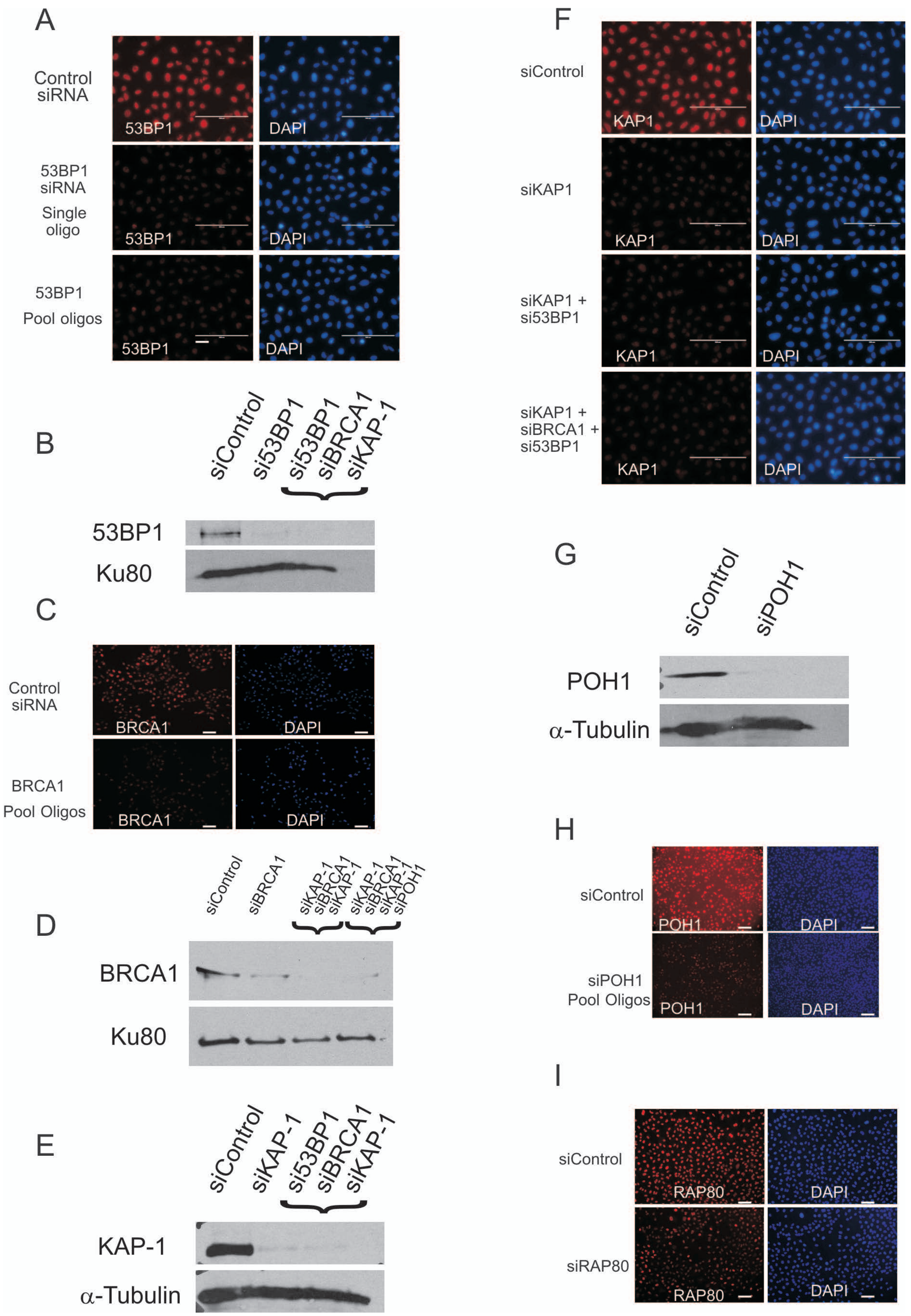

A
Control siRNA
53BP1
DAPI
53BP1 siRNA
Single oligo
53BP1
DAPI
53BP1
Pool oligos
53BP1
DAPI
B
siControl
si53BP1
si53BP1
siBRCA1
siKAP-1
53BP1
Ku80
C
Control siRNA
BRCA1
DAPI
BRCA1
Pool Oligos
BRCA1
DAPI
D
siControl
siBRCA1
siKAP-1
siBRCA1
siKAP-1
siKAP-1
siBRCA1
siKAP-1
siPOH1
BRCA1
Ku80
E
siControl
siKAP-1
si53BP1
siBRCA1
siKAP-1
KAP-1
α-Tubulin
F
siControl
KAP1
DAPI
siKAP1
KAP1
DAPI
siKAP1 +
si53BP1
KAP1
DAPI
siKAP1 +
siBRCA1 +
si53BP1
KAP1
DAPI
G
siControl
siPOH1
POH1
α-Tubulin
H
siControl
POH1
DAPI
siPOH1
Pool Oligos
POH1
DAPI
I
siControl
RAP80
DAPI
siRAP80
RAP80
DAPI

Figure S1

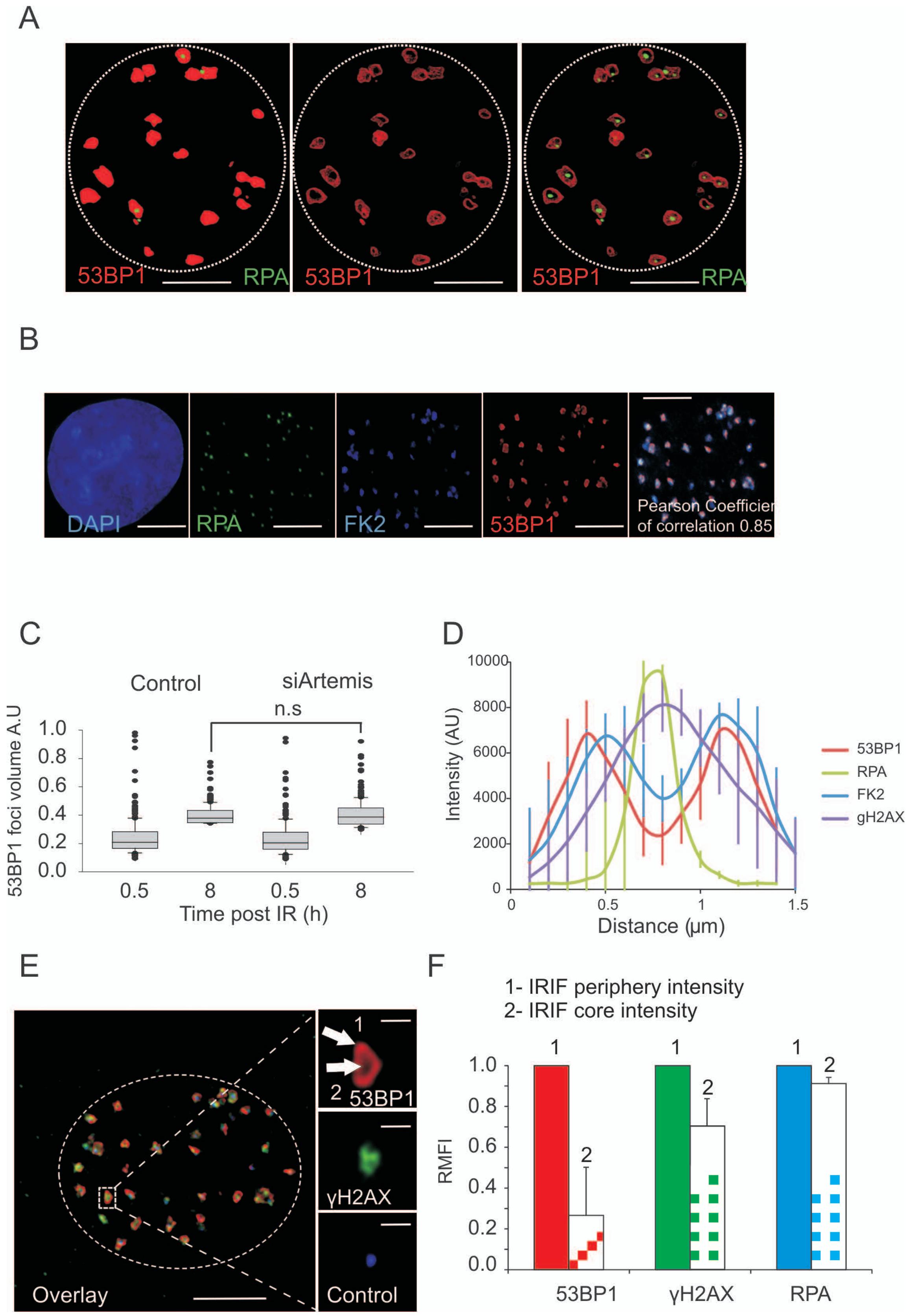
A
53BP1
RPA
53BP1
53BP1
RPA
B
DAPI
RPA
FK2
53BP1
Pearson Coefficien
of correlation 0.85
C
Control
siArtemis
n.s
53BP1 foci volume A.U
1.0
0.8
0.6
0.4
0.2
0.0
0.5
8
0.5
8
Time post IR (h)
D
Intensity (AU)
10000
8000
6000
4000
2000
0
0
0.5
1
1.5
Distance (μm)
53BP1
RPA
FK2
gH2AX
E
1
2 53BP1
γH2AX
Control
Overlay
F
1- IRIF periphery intensity
2- IRIF core intensity
RMFI
1.0
0.8
0.6
0.4
0.2
0.0
53BP1
γH2AX
RPA

Figure S2

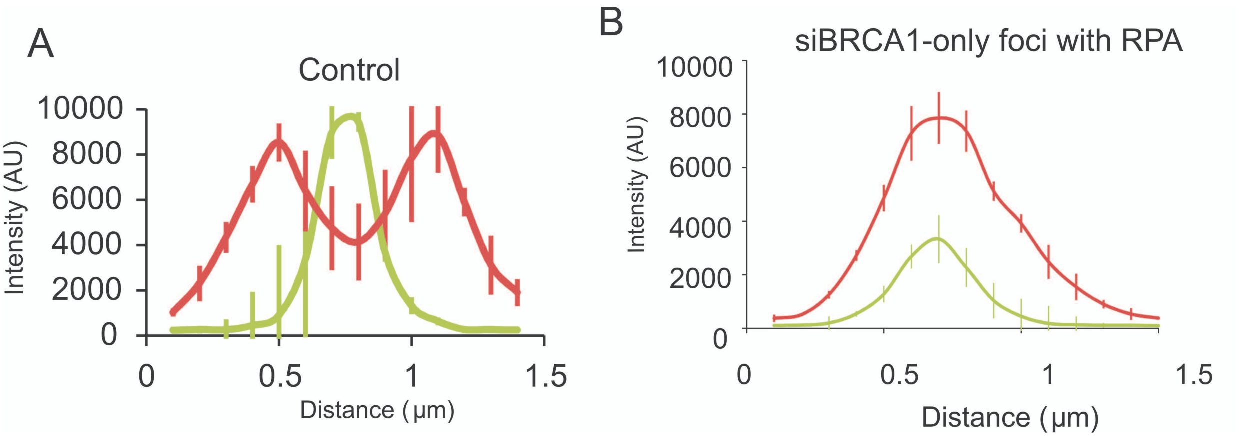
A
Control
10000
8000
6000
4000
2000
0
Intensity (AU)
0
0.5
1
1.5
Distance (µm)
B
siBRCA1-only foci with RPA
10000
8000
6000
4000
2000
0
Intensity (AU)
0
0.5
1
1.5
Distance (µm)

Figure S3

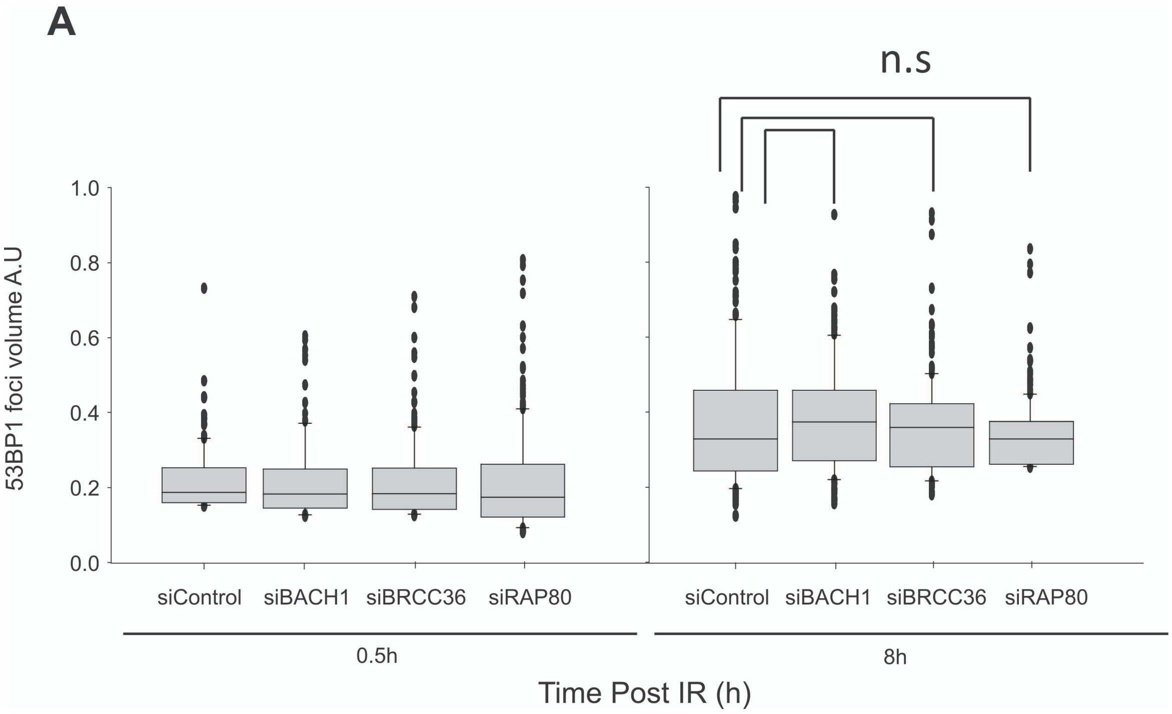
A
n.s
53BP1 foci volume A.U
1.0
0.8
0.6
0.4
0.2
0.0
siControl
siBACH1
siBRCC36
siRAP80
siControl
siBACH1
siBRCC36
siRAP80
0.5h
8h
Time Post IR (h)

Figure S4

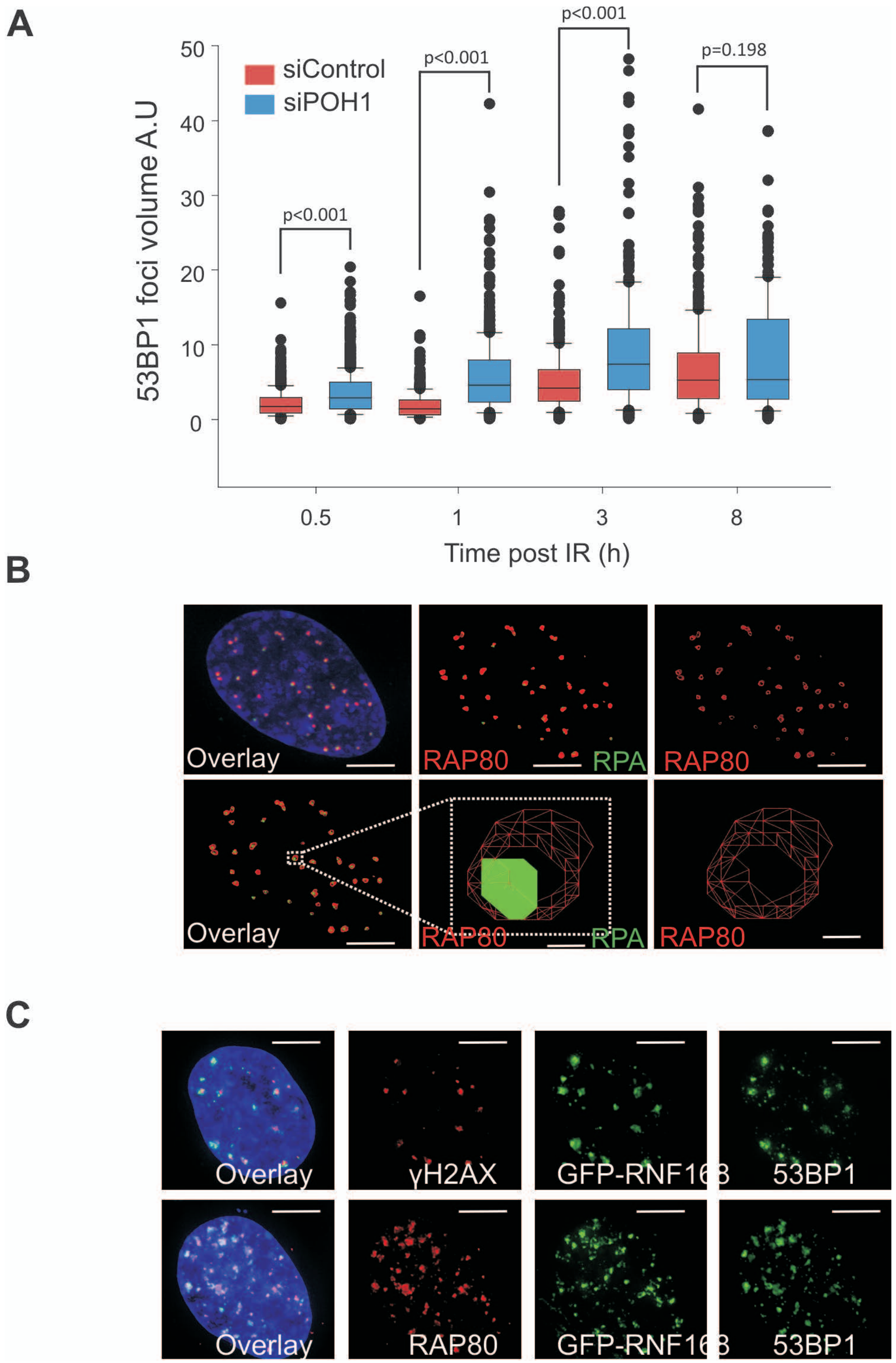
A
siControl
siPOH1
53BP1 foci volume A.U
50
40
30
20
10
0
p<0.001
p<0.001
p<0.001
p=0.198
0.5
1
3
8
Time post IR (h)
B
Overlay
RAP80
RPA
RAP80
Overlay
RAP80
RPA
RAP80
C
Overlay
γH2AX
GFP-RNF168
53BP1
Overlay
RAP80
GFP-RNF168
53BP1

Figure S5

A

	siControl				siBRCA1				siPOH1			
IRIF	53BP1	FK2	RAP80	RPA	53BP1	FK2	RAP80	RPA	53BP1	FK2	RAP80	RPA
Hole size (μM)	0.75	0.65	0.68	-				-	-	-	-	-
IRIF size (μM)	1.2	1	1.14	0.31	0.55	0.55	0.68	-	0.95	0.93	0.93	-
	siBRCA1 + si53BP1				si53BP1 + siBRCA1 + siKAP1				siPOH1 + siRAP80			
IRIF	53BP1	FK2	RAP80	RPA	53BP1	FK2	RAP80	RPA	53BP1	FK2	RAP80	RPA
Hole size (μM)	-	-	-	-	-	0.63	0.62	-	0.58	0.7	-	-
IRIF size (μM)	-	0.69	0.67	-	-	0.94	0.95	0.27	1.17	1.10	-	0.33
	siPOH1+ si53BP1 + siKAP1				si53BP1 + siBRCA1 + siKAP1 + siPOH1				siBRCA1 + siPOH1 + siRAP80			
IRIF	53BP1	FK2	RAP80	RPA	53BP1	FK2	RAP80	RPA	53BP1	FK2	RAP80	RPA
Hole size (μM)	-	-	-	-	-	-	-	-	-	-	-	-
IRIF size (μM)	-	1.15	1.2	-	-	0.63	0.63	-	0.58	0.61	-	-
	siRAP0				si53BP1 + siKAP1				siCtIP			
IRIF	53BP1	FK2	RAP80	RPA	53BP1	FK2	RAP80	RPA	53BP1	FK2	RAP80	RPA
Hole size (μM)	0.78	0.71	-	-	-	0.75	0.68	-	-	-	-	-
IRIF size (μM)	1.1	1.1	-	0.30	-	1.2	1.1	0.29	0.58	0.6	0.6	-

B

Target gene (Human)	Target sequence (5'-3')
BRCA1	CAACAUGCCCACAGAUCAA<br>CCAAAGCGAGCAAGAGAAU<br>UGAUAAAGCUCCAGCAGGA<br>GAAGGAGCUUUCAUCAUUC
BRCA2	GAAACGGACUUGCUAU UUA<br>GGUAUCAGAUGCUUCAUUA<br>GAAG AAUGCAGGUUUAAUA<br>UAAGGAACGUCAAGAGAUA
Artemis	CACCAAAGCUUUUCAGUGA<br>UGAAUAAGCUAGACAUGUU<br>GCACAACUAUGGAUAAAGU<br>GUACGGAGCCAAAGUAUAA
BRCC36	GCAUAUACUGGAACUGAAA<br>GAAGGACCGAGUAGAAAUU<br>CGUCAGAAUUGUUCACAUU<br>CAUAAUGGCUCAGUGUUUA
RAP80	GUAAAUCCCUGGUCCCAUU<br>AAAUGAAUCUCCCGUCAAG<br>AGAGCAGGCUAGUGAGAAA<br>AGAGGCAGCUCCUUAAUAA
POH1	GGCAUUAAUUCAUGGACUA<br>GAACAAGUCUAUAUCUCUU<br>AGAGUUGGAUGGAAGGUUU<br>GAUGGUUGUUGGUUGGUAU
KAP-1	GAGCAUAGAUACCAAUUUA<br>UAACUGUGCCUGAUUAUUA<br>GAAGAACGCCAGUUGCUUA<br>GAUCAUAGAUACACUAAUC
CtIP	GCACGUUGCCCAAAGAUUC<br>GAACAGAAUAGGACUGAGU<br>GAGGUUAUAUUAAGGAAGA<br>GGAGCUACCUCUAGUAUCA

Figure S6

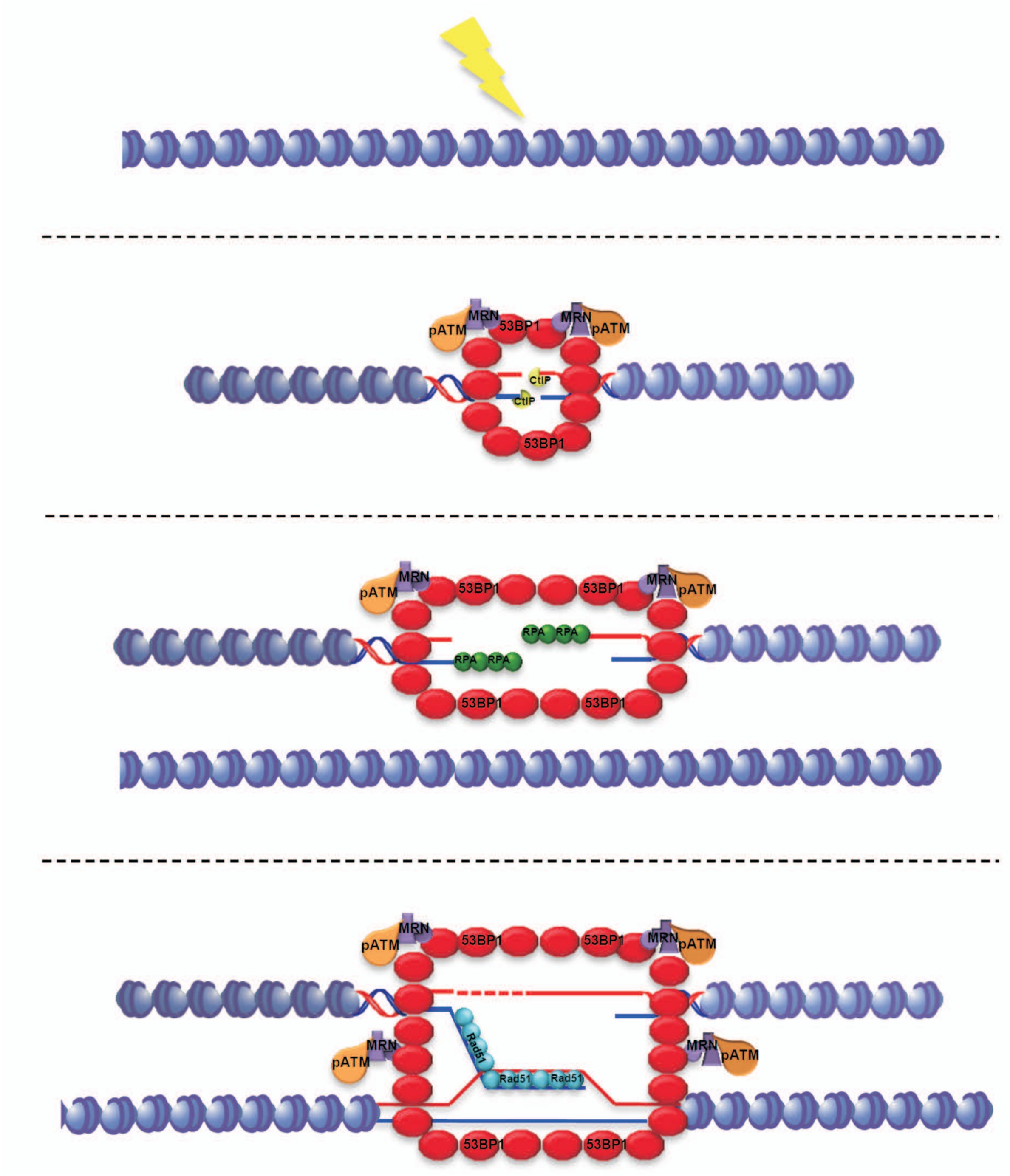

Figure S7
