# Supplementary material for: Co-operation of BRCA1 and POH1 relieves the barriers posed by 53BP1 and RAP80 to resection
Source: Nucleic Acids Res. 2013 Sep 5;41(22):10298–311. doi: 10.1093/nar/gkt802 (PMC3905848; doi:10.1093/nar/gkt802)
Supplement: Supplementary Data [file supp_gkt802_nar-00526-d-2013-File002.docx]

**Supplementary Figure 1: Controls for siRNA knockdown efficiencies.**

The indicated siRNA duplexes were transfected into 2 x 10^5^ logarithmically growing cells for each knockdown. Cells were then grown for 72 h prior to fixation and immunostaining/immunoblotting with the indicated antibodies. A) Knockdown efficiency in A549 cells following treatment with either a single or a pool of 53BP1 siRNA oligonucleotides (the single oligonucleotide was distinct to any in the pool). Scale bars- 200μm. B) 53BP1 knockdown efficiency when using the single 53BP1 siRNA oligonucleotide either alone or in combination with other siRNA oligonulceotides. Knockdown efficiency was not significantly affected by the simultaneous transfection of other siRNA oligonucleotides.Ku80 was used as a loading control.

C-D) BRCA1 pool and single oligonucleotide knockdown efficiency was assessed by immunofluorescence (C) and Western Blotting (D). BRCA1 knockdown efficiency using the single BRCA1 siRNA oligonucleotide alone or in combination with other siRNA oligonucleotides was assessed by Western Blotting (D). BRCA1 knockdown efficiency was not significantly affected by simultaneous transfection of other siRNA oligonucleotides. Ku80 was used as a loading control.

E, F) KAP-1 knockdown efficiency was assessed using a KAP-1 siRNA oligonucleotide either alone or in combination with other siRNA oligonucleotides by Western Blotting (E) and immunofluorescence (F) . KAP-1 knockdown efficiency was not significantly affected by the simultaneous transfection of other siRNA oligonucleotides. α-Tubulin was used as a loading control.

G-H) POH1 knockdown efficiency was assessed following treatment with either a single or a pool of POH1 siRNA oligonucleotides (the single oligonucleotide was distinct to any in the pool) by Wesetern Blotting (G) or immunofluorescence (H).

I) RAP80 knockdown efficiency was assessed following treatment with a pool of RAP80 siRNA oligonucleotides by immunofluorescence.

**Supplementary figure 2**: **53BP1 is repositioned together with FK2 IRIF to allow RPA foci formation, via an active process that is not a consequence of resection.**

A) 3D model of G2 phase A549 cell harvested 8h post 3 Gy IR and immunostained with the indicated antibodies. The left panel is a ‘solid’ representation of both 53BP1 and RPA signals. In the middle and right panels, 53BP1 signal is shown as ‘wireframe’ depicting the hollow center of 53BP1 foci where RPA foci form. B) A549 cells were immunostained with the indicated antibodies 8 h post 3Gy IR and colocalization between 53BP1 and FK2 was carried out using softWoRx® Suite software. The Pearson Coefficient of Correlation indicates how closely the two intensities are colocalized on a pixel-by-pixel basis (full colocalization is 1.0). Scale bars- 5μm.

C) 53BP1 foci volume estimations following siRNA Artemis. Analysis was undertaken as in Fig. 1D. 53BP1 foci volume at 8h post 3Gy IR in Artemis depleted cells was normal despite these cells being defective in resection. This indicates that 53BP1 foci enlargement in G2 phase is an active process rather than a consequence of ongoing resection.

D) Quantification of fluorescence intensity profiles along a line drawn through the centre of IRIF at 8h post irradiation. By 8 h post IR, 53BP1 and FK2 IRIF are distributed in a bipolar manner. No change in the distribution of gH2AX could be detected by this procedure. A minimum of 10 cells were analysed from each of three independent experiments. Error bars represent SD. E, F) Comparison of 53BP1, γH2AX and RPA intensity in the periphery and core of IRIF. Due to variation in the shape of the devoid core between cells, we did not observe a bimodal distribution of gH2AX in panel D. However, imaging analysis did reveal the presence of a devoid core region, albeit smaller than that observed for 53BP1. As a more sensitive method to quantify changes in protein intensity in IRIF, we assessed the intensity in small, circular regions either on the IRIF periphery (1) (for each protein) or within the IRIF core (2). The IRIF core was determined as the region where RPA foci form.Panel F shows the quantification of the intensity measurements obtained by the softWoRx® Suite software and plotted as the intensity in the IRIF core relative to the intensity on the IRIF periphery. Measurements were obtained from a minimum of 50 IRIF, in several different cells, from two independent experiments. Error bars represent SD.

**Supplementary Figure 3. Analysis of the intensity of RPA at IRIF following siRNA BRCA1.**

A) In control cells, the intensity of RPA along a track taken through the IRIF revealed a monomodal distribution of RPA as shown. Data shown is the same as that in Figure 3D.

B) Following siRNA BRCA1, ~ 50% of IRIF harboured RPA foci and the remaining foci showed no evident RPA. In panel B, the intensity of RPA was measured in those IRIF that had visible RPA foci. This demonstrates a reduced intensity of RPA in those IRIF although they appeared similar in size by normal microscopy. The combined analysis involving the averaging of all IRIF following siRNA BRCA1 is shown in Figure 2E.

Taken together with the data in Figure 1, these findings show that following siRNA BRCA1, the number of RPA foci is only reduced ~ 2 fold. By normal microscopy the size of the RPA foci that form appear normal. However, using the Delta Vision microscope and intensity analysis, the level of RPA at the IRIF is diminished. These findings argue that BRCA1 is required for efficient resection. To explain these findings, we propose that RPA foci formation requires a defined threshold level of resection; below this level of resection, RPA foci fail to form whilst above a critical level of resection, RPA can form on the entire length of ssDNA. A subtle difference in the quality of RPA at the IRIF is likely due to the fact that further elongation of resection is impaired following siBRCA1. The threshold level of resection could be ~ 25 bp given that this is the length of ssDNA required to activate ATR. This threshold effect explains the apparent “all or none” presence of RPA in conditions that impair but do not abolish resection. Significantly, the number of RAD51 foci is more substantially impaired following siRNA BRCA1 consistent with the notion that the quality of the RPA foci that do form is impaired.

**Supplementary Figure 4. The BRCA1 interacting proteins BACH1, RAP80 and BRCC36 are dispensable for 53BP1 repositioning during HR.**

Analysis 53BP1 foci volume in A549 cells treated with the indicated siRNA. The cells were irradiated with 3Gy and harvested at 0.5h and 8h post IR. The volume of 53BP1 foci was assessed by 3 dimensional imaging using an Applied Precision® Delta Vision® RT Olympus IX70 deconvolution microscope and softWoRx® Suite software at the indicated times (see materials and methods for details of image processing).

**Supplementary Figure 5: 53BP1 foci enlarge more rapidly following siPOH1 in G1 phase, but are not significantly larger by 8h post IR.**

Analysis 53BP1 foci volume in G1 phase A549 cells treated with the indicated siRNA. The cells were irradiated with 3Gy and harvested from 0.5h to 8h post IR. The area of 53BP1 foci was assessed by analyzing images acquired using an Applied Precision® Delta Vision® RT Olympus IX70 deconvolution microscope with the softWoRx® Suite software. In each sample a minimum of 10 cells were analyzed from three independent experiments. Statistical analysis was carried out using the Mann Whitney Rank Sum test. Data were deemed to be significant when a p value < 0.05 was obtained. B) 3D model of G2 phase A549 cell harvested 8h post 3 Gy IR and immunostained with the indicated antibodies. The top two panels are a ‘solid’ representation of both RAP80 and RPA signals. In the top right panel and in the lower panels, RAP80 signal is shown as ‘wireframe’ depicting the hollow center of RAP80 foci where RPA foci form. Scale bars- 5μm. C) A549 cells transiently expressing GFP-RNF168 were irradiated with 3Gy, and 8 h post IR, fixed and stained with the indicated antibodies. Since over expression of RNF168 has been reported to result in enlarged 53BP1 foci, we only analysed IRIF where the size of 53BP1 foci appeared similar to those in control cells. In these cells, we observed that RNF168 overlapped with 53BP1 and RAP80 and hence appeared also to be repositioned. IRIF with enlarged 53BP1 (compared to cells not transfected with RNF168-GPF) at 8 h post IR were also observed but were not examined.Scale bars- 5μm.

**Supplementary Figure 6: Quantification of IRIF clearance and overall size in cells treated with combined siRNA.**

A) Quantification of IRIF clearance and overall size in cells treated with the indicated siRNA oligos. IRIF clearance (hole size) represents the distance between the peaks in IRIF that show a bipolar distribution, while the foci size was determined by measuring the distance between the outer edges of the peaks at 50% intensity.

B) Full list of target sequences from the Dharmcacon ON-TARGETplus SMARTpool,

**Supplementary Figure 7**: **Model showing the expansion of 53BP1 foci in G2 phase and the formation of an IRIF core devoid of 53BP1 and FK2 where RPA foci form.**

Model showing the requirement for 53BP1 to affect HC changes on the undamaged sister chromatid. The slowly repaired DSBs that arise within regions of heterochromatin (HC) undergo resection and repair by HR (HC is depicted in green). In early G2 phase (30 min post IR), 53BP1 foci form around the DSB on a single DNA molecule similar to the situation in G1 phase. 53BP1 is likely not located at the extreme DNA end. 53BP1 interacts with RAD50 of the MRN complex; NBS1 of the MRN complex interacts with ATM. Thus, ATM tethering at the DSB is enhanced by the presence of 53BP1 foci (interactions between MRN and MDC1 or H2AX also enhance ATM tethering but 53BP1 appears to be critical for the presence of pATM foci at DSBs). ATM phosphorylates KAP-1 located at HC-DSBs in a concentrated manner producing pKAP-1 foci (note that pan-nuclear pKAP-1 also occurs at early times post IR but this is not 53BP1-dependent; in contrast the formation of pKAP-1 foci, which only form at HC-DSBs is 53BP1-dependent). This causes HC-relaxation in the vicinity of the DSB (not depicted in the figure). HC forms a partial barrier to resection that is overcome by 53BP1-ATM pKAP-1 foci formation. The presence of 53BP1 on the DNA molecule also forms a partial barrier to the completion of resection and a bigger barrier to RAD51 loading. However, neither 53BP1 nor KAP1 prevent the initiation of resection by CtIP-MRN, since they function downstream of CtIP-MRN dependent commitment to HR and since RPA foci numbers are only partially reduced. Later in G2 (evident at 2 h), further resection ensues followed by the single stranded DNA overhang with bound RPA and/or RAD51 invading the undamaged sister template. 53BP1 foci formation expands to allow further resection and to encompass the undamaged DNA molecule, resulting in a two fold expansion in foci volume. The expansion of 53BP1 foci requires the BRCT but not the RING finger domain of BRCA1 and involves repositioning of ubiquitin modifications on histones. Thus, we propose that BRCA1 promotes deubiquitination and/or proteasome mediated protein degradation in the core region and new ubiquitin events on the damaged and, we propose, the undamaged molecule. For simplicity this has not been depicted in the figure. The presence of 53BP1 on the undamaged strand allows pKAP1 formation and hence HC relaxation on this strand, which promotes the completion of resection and/or RAD51 loading. We have depicted 53BP1 as entirely encircling the DNA molecule although its initial tethering involves interactions with methylated H4. This is consistent with evidence that 53BP1 undergoes oligomerisation and has a role in synapsis during long range V(D)J recombination (Adams et al, 2005; Difilippantonio et al, 2008). In G2 phase, 53BP1 may also enhance synapsis but this does not appear to be essential since HR can ensue in the absence of 53BP1+BRCA1. We suggest that the close association of sister chromatids in G2 phase via cohesin interactions can function redundantly to 53BP1-dependent synapsis in G2 phase. Thus, 53BP1-dependent synapsis is only essential for long-range translocation events in G1 phase.
